# Presuppositions of determiners are immediately used to disambiguate utterance meaning: A mouse-tracking study on the German language

**DOI:** 10.1007/s00426-020-01302-7

**Published:** 2020-04-04

**Authors:** Cosima Schneider, Nadine Bade, Michael Franke, Markus Janczyk

**Affiliations:** 1 University of Tübingen, Schleichstrasse 4, 72076 Tübingen, Germany; 2 Institut Jean Nicod, Département d’études cognitives, ENS, EHESS, PSL University, CNRS, 29 rue d’Ulm, Paris, France; 3 Osnabrück University, Wachsbleiche 27, 49090 Osnabrück, Germany; 4 University of Bremen, Hochschulring 18 (Cognium), 28359 Bremen, Germany

## Abstract

The present study investigated how listeners understand and process the definite and the indefinite determiner. While the definite determiner clearly conveys a uniqueness presupposition, the status of the anti-uniqueness inference associated with the indefinite determiner is less clear. In a forced choice production task, we observed that participants make use of the information about number usually associated with the two determiners to convey a message. In a subsequent mouse-tracking task, participants had to select one of two potential referents presented on screen according to an auditorily presented stimulus sentence. The data revealed that participants use the information about uniqueness or anti-uniqueness encoded in determiners to disambiguate sentence meaning as early as possible, but only when they are exclusively faced with felicitous uses of determiners.

**Electronic supplementary material:**

The online version of this article (10.1007/s00426-020-01302-7) contains supplementary material, which is available to authorized users.

## Introduction

Imagine a situation where someone says “The fridge at work is broken”. There are probably two assumptions you make when hearing this utterance. First, that there actually exists a fridge at the workplace of the speaker, and second, that there is exactly one fridge. Both of these assumptions are called presuppositions in the semantics/pragmatics literature and they are referred to as the presuppositions of existence and uniqueness of the definite determiner “the”.

Presuppositions are background assumptions that speakers of a conversation take to hold. They are usually triggered by a lexical item, the so-called presupposition trigger. In the example above, this would be the definite determiner “the”. Since we know that it can only be felicitously used with unique objects due to its presuppositions, we can draw conclusions about the number of fridges based on its use. Compare the example with the utterance “A fridge at work is broken.”, that is, with the same utterance containing the indefinite determiner “a”. The assumption about the existence of a fridge remains the same. However, there is no inference of the fridge being unique. Rather, one could deduce that there is actually more than one fridge at the workplace of the speaker. Similarly to the case of the definite determiner, we deduce this based on the fact that indefinite determiners are only felicitously used with non-unique objects. However, the status of this *anti-uniqueness* inference is more controversially discussed in the literature.^1^ It has been argued that both uniqueness and anti-uniqueness inferences are part of the non-literal meaning component of sentences. However, open questions are whether they are equal in strength, and how and when these different inferences arise.

The question we address in this paper is whether listeners use inferences associated with definite and indefinite determiners to disambiguate utterance meaning as early as possible, even if the speaker does not always use these determiners felicitously. Much previous research has provided evidence that listeners may, often swiftly, adapt to the idiosyncrasy of the given speaker. This applies to many different aspects of the interpretation of language, including phonetics/phonology (Kleinschmidt & Jaeger, 2015; Roettger & Franke, 2019), syntax (Fine, Jaeger, Farmer & Qian, 2013; Jaeger & Snider, 2013), semantics (Yildirim, Degen, Tanenhaus, & Jaeger, 2016), and pragmatic factors (Grodner & Sedivy, 2011; Stranahan, 2018). However, to the best of our knowledge, there is so far no work investigating adaptation effects of presuppositional information for online processing. Moreover, there is a vast amount of literature on the processing of felicitous and infelicitous uses of presuppositions in different contexts (see Schwarz 2007; Schwarz & Tiemann, 2012; Tiemann et al., 2011; Tiemann, 2014; for a recent review, see Schwarz, 2016), but almost no experimental investigations of when and how the interpretation of utterances may be affected by information encoded in the presuppositions of certain expressions. The present study tries to fill these gaps. To this end, we will compare how listeners’ online interpretation of definite and indefinite determiners changes depending on how reliable the speaker is in using the determiner felicitously. To do so, we compare a group of participants encountering only felicitous uses of determiners with a group that also encounters infelicitous uses of determiners. In the following, we will introduce presuppositions and the triggering mechanism in more detail, focusing on determiners. This is followed by a discussion of earlier work suggesting that the process of presupposition evaluation is started immediately on the presupposition trigger. We report one experiment comprising two parts: in a production task, we test whether participants use the presupposition usually associated with the determiner to convey information. The subsequent mouse-tracking task addresses whether participants use determiners to disambiguate utterance meaning and if so, whether this is different for the definite and the indefinite determiner.

### Presuppositions

Informally speaking, presuppositions are background assumptions that are shared by all interlocutors of a conversation. They are introduced by certain words, so-called presupposition triggers. One classical example of such a trigger is the definite determiner. It introduces the presupposition that there exists a unique individual with the property described by the noun it combines with. Technically speaking, these words introduce appropriateness conditions, that is, certain restrictions on what the context must look like for the sentence containing them to be felicitously uttered. The assumption is that if the presupposition of a trigger is not met in the context, uttering a sentence containing it is infelicitous. This is illustrated in (1). In (1-a), the presupposition of the definite determiner, that there is a unique apple, is fulfilled in the context. Thus, the sentence is felicitous. In (1-b), the presupposition of uniqueness is not fulfilled in the context and the sentence is infelicitous (or inappropriate).

1.
  - Context: There is an apple and a banana on the desk.
    Please give me the apple.
  - Context: There are three apples and a banana on the desk.
    # Please give me the apple.

Whereas felicitous uses of the definite determiner require a unique discourse referent (i.e., the definite determiner presupposes uniqueness), the indefinite determiner is assumed to require there to be more than one referent in the context. As a result, its use becomes odd if it is known that the referent is unique, as in (2).

2. # A sun is shining.

It was thus suggested in the literature that the indefinite determiner presupposes anti-uniqueness (see Kratzer, 2005, or the discussion in Heim, 1991, 2011) in the same way the definite determiner presupposes uniqueness. Under this view, both definite and indefinite determiners come with their own restrictions on what are appropriate contexts. Henceforth, we will refer to this theory as the “presupposition theory” and assume that both inferences are equally robust and accessed quickly.

However, Heim (1991) noted that (3) can be uttered without it being certain that there is more than one 20-ft-long catfish: it suffices that the speaker is not sure that there is exactly one 20-ft-long catfish.

3. Robert caught a 20-ft-long catfish.

From such observations, Heim (1991) concluded that the inference associated with the indefinite determiner is weaker than the presupposition of the definite determiner. To capture this, she proposed to add another principle to the Gricean maxims of conversation, Maximize Presupposition, which says: Presuppose as much as possible! (see also Chemla, 2009; Percus, 2006; Sauerland, 2008; Schlenker, 2012, for more refined versions of Maximize Presupposition). This principle can account for the fact that using indefinite determiners is infelicitous when it is common ground that the referent is unique, as in (4-a). More specifically, it explains the oddness of (4-a) by means of pragmatic reasoning over presuppositional stronger alternatives (Heim, 1991; Percus, 2006; Sauerland, 2008; Schlenker, 2012). The sentence in (4-b) is an alternative since it only differs from its competitor regarding the presuppositions it introduces. It introduces more presuppositions that are true in the context (since we know people have one unique father) and is thus the presuppositionally stronger alternative. When a listener hears the presuppositionally weaker sentence (4-a), s/he assumes the speaker must believe the presupposition of the stronger alternative to be false. The reasoning behind this conclusion is based on two main assumptions: (1) that the speaker obeys the conversational maxims including Maximize Presupposition, and (2) that the speaker tries to be cooperative in doing so. The hearer thus assumes that if the speaker believed the presupposition of (4-b) to be true, s/he would have used this version, because it would be more informative on a presuppositional level. Since s/he did not, s/he must believe it to not hold. The belief that the victim does not have a unique father, however, is contrary to common knowledge and, therefore, leads to the oddness of (4-a).^2^

4.
  - $*$ A father of the victim arrived at the crime scene. (Heim, 1991)
  - The father of the victim arrived at the crime scene.

In other words, anti-uniqueness is derived by (1) considering the (stronger) alternative with the definite determiner and (2) negating its presupposition. The inferences, which are the result of pragmatic reasoning based on Maximize Presupposition, are not presuppositions proper under this view. Henceforth, we will refer to them as anti-presuppositions (Percus, 2006). They are theoretically kept apart from presuppositions (and implicatures) and should be processed differently. As opposed to the assumptions of the “presupposition theory” introduced above, the anti-presupposition arising with the indefinite determiner has a weaker status than the presupposition of the (stronger) definite determiner; we will refer to this theory as the “anti-presupposition theory” in the following. Because the anti-uniqueness inference is derived by initially considering the (stronger) alternative of the definite determiner and subsequently negating it, processing of the indefinite determiner should thus be more complex than processing the definite determiner.

A third type of theory assumes that the indefinite determiner triggers an implicature due to its competition with other quantificational terms, for example, “every/all” or “another” (Chierchia, Fox, & Spector, 2012; Grønn & Sæbø, 2012). These quantificational terms form a lexical scale with the indefinite determiner (Horn, 1972). An implicature arises when the weaker item on such a scale is chosen, in this case the indefinite determiner. All items that are higher in the scale (items that trigger stronger alternatives) get negated: for example, the implicature of “A boy came” is that “Not all boys came”. This negation process requires the assumption of existence of other boys, and anti-uniqueness follows as a consequence. Contrary to the competition on a presuppositional level according to Maximize Presupposition, the competition between alternatives in this case arises on the level of assertion (“a” and “all” differ on the level of assertion, whereas “a” and “the” are alike on that level). Variants of this argue that anti-presuppositions *are* essentially implicatures in that they can be informative and follow from the same general mechanism (of exhaustification) (Magri, 2009; Schlenker, 2012; Singh, 2011). According to these theories, the definite and indefinite determiners are also asymmetric in the inferences they introduce: whereas the indefinite determiner should come with an implicature, which has shown to be processed even more rapidly (at least if certain conditions are met) than presuppositions (Bill, Romoli, & Schwarz, 2018; but see also Chemla, 2008), the definite determiner should come with a presupposition. We will refer to this theory as the “implicature theory”.

In sum, three different approaches are available to explain the effects of uniqueness and anti-uniqueness resulting from the definite and the indefinite determiner. (1) According to the “presupposition theory”, both determiners carry their own presuppositions proper and thus processing the two determiners should be equally difficult. (2) According to the “anti-presupposition theory” based on Maximize Presupposition, the anti-uniqueness inference of the indefinite determiner is derived indirectly from negating the uniqueness presupposition of the definite determiner. Thus, processing indefinite determiners should be more difficult than processing a definite determiner. (3) According to the “implicature theory”, the indefinite determiner comes with an implicature instead of an (anti-)presupposition. In this case, one may expect the indefinite determiner to be more easily processed than the definite determiner.

### Previous investigations of processing determiners

Experimental investigations of presuppositions have increased in recent years (for a recent review, see Schwarz, 2016). Here, we focus on studies dealing with (1) the early processing of presuppositions/inferences triggered by determiners, (2) differences between felicitous and infelicitous uses of determiners, and (3) potential differences between definite and indefinite determiner.

In a self-paced reading study, Altmann and Steedman (1988) investigated the syntactic consequences of a definite noun phrase having its presuppositions met or not met by the context. The data revealed early processing of the presupposition, before the end of the sentence. More precisely, participants were presented with test sentences in two different contexts. Context 1 introduced two candidates for a potential referent (a safe with a new lock and a safe with an old lock, see (5)), while Context 2 introduced exactly one candidate for a potential referent (see (6)).

5. Context 1: A burglar broke into a bank carrying some dynamite. He planned to blow open a safe. Once inside he saw that there was a safe with a new lock and a safe with an old lock.
6. Context 2: A burglar broke into a bank carrying some dynamite. He planned to blow open a safe. Once inside he saw that there was a safe with a new lock and a strongbox with an old lock.

In the test sentence in (7), the prepositional phrase “with the new lock” modifies the noun phrase “safe”. As a result, the uniqueness presupposition is met in both contexts for this test sentence. This is not the case for the test sentence in (8), whose presupposition is only satisfied by Context 2.

7. The burglar/blew open/the safe/with the new lock/and made of/with the loot.
8. The burglar/blew open/the safe/with the dynamite/and made of/with the loot.

Reading times differed in the disambiguation region (i.e., on the prepositional phrase “with the new lock” or “with the dynamite”). Test sentences with an unmet uniqueness presupposition as in (8) were read slower than test sentences as in (7). Thus, the authors conclude that people experience processing difficulties at an early point in time, when the uniqueness presupposition of the definite determiner is not met. However, no evidence for processing difficulties on the presupposition trigger itself was reported. We believe that the relatively late effects are most likely due to the experimental design. In particular, the content of the presuppositions was only known on the prepositional phrase, because only then it was clear which referent was considered unique in the context. In sum, the results suggest that a presupposition is processed as soon as it is fully known. A more detailed analysis of syntactic ambiguity resolution strategies was done by Spivey, Grosjean, and Knoblich (1995) who provide further evidence for an immediate influence of pragmatics and logically specific biases in syntactic ambiguity resolution.

Tiemann et al. (2011) reported three self-paced reading studies and acceptability ratings on presupposition processing as induced by different triggers (German “wieder”: English “again”; “auch”: “also”; “aufhören”: “stop”; “wissen”: “know”, and definites in the shape of possessive noun phrases). In their first experiment, which is important for the current paper, they focus on the processing of the trigger itself. Participants were presented with a context (as in (9)) and the authors compared test sentences including a presupposition trigger (as in (10)) with sentences including a neutral word that does not trigger a presupposition (as in (11)), and with semantically unacceptable sentences (as in (12)).

9. Context: Tina ist mit einer guten Freundin shoppen.
  Tina is shopping with a good friend.
10. Sie kauft wieder rote Handschuhe.
  She buys red gloves again.
11. Sie kauft heute rote Handschuhe.
  She buys red gloves today.
12. *Sie kauft freundlich rote Handschuhe.
  She buys red gloves friendly.

Sentences with the neutral word were rated best, followed by sentences including the presupposition trigger and unacceptable sentences. Second, and most importantly, reading time data revealed that—for the position of the presupposition trigger—sentences with a trigger induced the longest reading times, followed by sentences with a neutral word, while unacceptable sentences were read fastest (see also Schneider, Bade, & Janczyk, 2020). This pattern suggests that a presupposition trigger immediately demands more attention, because it alerts the reader to consider the preceding context. Furthermore, in their third experiment, they investigated whether a presuppositional sentence in a neutral context (neither making the presupposition explicitly true nor false) evokes longer reading times than in a falsifying or verifying context. The data revealed early effects on the trigger, which also suggest that processing of the presupposition begins immediately upon encountering the trigger.

Unfortunately, reading times were not analyzed for individual presupposition triggers, as there were not enough items for each trigger to allow for strong conclusions. It is thus unclear whether the same pattern holds for all triggers or not.

There is further evidence for an immediate processing of presupposition triggers from electrophysiological studies. For example, van Berkum, Brown, and Hagoort (1999) and van Berkum, Brown, Hagoort, and Zwitserlood (2003) used event related potentials (ERPs) to investigate the interplay of referential and structural factors during sentence processing in discourse. To do so, the authors used referentially ambiguous noun phrases. In these studies, participants were presented with a discourse as in (13) and (14). A corresponding test sentence was, for example, “David told the girl that had been on the phone to hang up.” In discourse (13), uniqueness of the noun phrase “the girl” in the test sentence was granted, because the discourse introduces only one salient girl. In contrast, this is not the case in the other discourse (14), where both girls are equally salient. As a result, the uniqueness presupposition is not fulfilled up to the disambiguating relative clause (“...that had been on the phone...”).

13. David had told the boy and the girl to clean up their room before lunchtime. But the boy had stayed in bed all morning and the girl had been on the phone all the time.
14. David had told the two girls to clean up their room before lunchtime. But one of the girls had stayed in bed all morning and the other girl had been on the phone all the time.

The definite noun phrases evoked early ERP effects already on the noun, when the uniqueness presupposition was not met. Thus, referential ambiguity appears to be detected very early during sentence processing. Contrary to Altmann and Steedman (1988), evaluation of the presupposition was not delayed until to the relative clause, but instead participants considered the presupposition against the context as early as they heard or read the noun. This may come as a surprise given that participants knew that there were also sentences where the presupposition failure was resolved by the following relative clause, similar to the participants in Altmann and Steedman (1988) becoming aware that the following prepositional phrase was important for evaluating the presupposition. The difference might be due to the different syntactic status of relative clauses and prepositional phrases. Taken together, the studies of van Berkum et al. (1999, 2003) support the idea of early processing of presuppositions triggered by the definite determiner. Participants realize that there might be presupposition failure of a definite noun phrase immediately on the noun itself.

Evidence for immediate presupposition processing also comes from Kirsten et al. (2014) who investigated the processing of definite and indefinite determiners in an ERP study. Two types of contexts (see (15)) introduced either a single referent (e.g., one polar bear) or multiple referents (e.g., some polar bears). Test sentences were alike except for the determiner used (“the/a”) and were either presented in a matching condition where the context sentence introduced the noun phrase with an indefinite determiner “ein/e” (Engl.: “a”) or in a mismatching condition where it contained a quantifier such as “einige” (Engl.: “some”) or “viele” (Engl.: “many”).

15.
  - Antje war gestern im Zoo in Düsseldorf und besuchte einen /einige Eisbären im Bärengehege.
  - Antje visited the Düsseldorf zoo yesterday and saw a/some polar bear/s in the bear enclosure.

16.
  - Antje beobachtete, dass der/ein Eisbär sehr aggressiv war.
  - Antje noticed that the/a polar bear was very aggressive.

The data revealed that participants recognized mismatching conditions already when reading the determiner. For both determiners, the mismatching effect became visible through an N400 and a P600 effect after onset of the noun.^3^ Thus, the results support the idea of immediate processing of presuppositions, already starting on the trigger.

There is further evidence that information encoded in determiners is exploited to guide behavior. Dahan, Swingley, Tanenhaus, and Magnuson (2000) focused on gender information encoded in the determiner (in the French language) in an eye-tracking study and demonstrated that gender-marked determiners immediately directed the listeners’ eyes towards the object that matched the gender. This supports the idea of immediate processing of determiners and the use of information encoded in therein.

Further support for early processing of determiners comes from a visual-world eye-tracking study using a picture selection task (Bade & Schwarz, 2019a). In one critical condition, sentences like “A/The shirt in Benjamin’s closet is blue” were presented auditorily and paired with three different pictures, all of which showed a boy with a closet. On one of the pictures, the closet contained three shirts, one of which was blue (non-unique condition). On another picture, only one blue shirt was depicted (unique condition), and no shirts were depicted on a third (distractor) picture. Participants were asked to choose the picture they thought was corresponding to the sentence, and indeed they looked at the respective target picture (picture with a single shirt for the definite determiner, and picture with multiple shirts for the indefinite determiner) very early on upon hearing the noun. This suggests that the (anti-)uniqueness information encoded in the determiners was used rapidly for interpretation. In addition, differences between both determiners were observed. Inferences based on the use of the indefinite determiner were drawn to a much lesser degree than those evoked by the use of the definite determiner, as demonstrated by fewer target choices for the indefinite than for the definite determiner. Further differences between the determiners were observed in eye-tracking patterns for the cases where the target was chosen. Overall, these results are in line with the “anti-presupposition theory”, which predicts differences in processing patterns associated with the anti-uniqueness inference and the uniqueness presupposition.

Finally, mouse-tracking data from Schneider et al. (2019) also support this view. In two experiments, participants were asked to judge the appropriateness of sentences like “Of these, Jan received the/a banana.” in contexts showing that Jan’s mother bought three pieces of fruit (e.g., one banana and two pears, or two bananas and one pear). Participants made their judgment by moving the mouse cursor into response boxes located in the top right and left corners of the computer screen. The indefinite determiner was associated with more difficulty in processing (reflected in longer movement times and a larger area under the curve). Most importantly, the data of Schneider et al. (2019) also revealed an initial deviation into the direction of the non-target response (i.e., the competitor) for the indefinite determiner. This was predicted by the “anti-presupposition theory” exploiting Maximize Presupposition (Heim, 1991), which suggests that participants first consider the uniqueness presupposition of the definite alternative when encountering an indefinite determiner.

In sum, there is evidence for early processing of presuppositions introduced by definite determiners, as demonstrated by effects observed on the trigger and briefly thereafter, when the content of the presupposition is known. Furthermore, additional processing costs were observed when the presupposition is not met by the context. This effect may, however, be modulated by additional cognitive load (see Clifton, 2013). Finally, the available evidence suggests processing differences between determiners.

Overall, the existing experimental literature on presuppositions focused on the processing costs associated with presuppositions of different triggers in different contexts. One question that this research concentrated on was whether presuppositions are more difficult to process in contexts in which their use is infelicitous (i.e., when the presupposition was not verified). Another focus was on the question, when these effects occur. With the exception of Bade and Schwarz (2019a, b) previous research did not address the issue of whether early information about uniqueness and anti-uniqueness encoded in determiners is used to make predictions about a sentence’s meaning. A weakness of the few studies that addressed how inferences associated with definite or indefinite determiners affect interpretation (Bade & Schwarz, 2019a) is that it was unclear from the task used in the experiment whether the information encoded by the determiner would be relevant for the choice. In general, it was unclear for the participants what was hinging on their choice of picture. Another weakness of many experiments on definiteness in general is that it only became apparent on the noun whether uniqueness or anti-uniqueness was satisfied, because more than one referent to which the definite or indefinite noun phrase could refer was provided in the context.

The experiment reported in the present paper extends the still small empirical basis for answering the following question: Is interpretation driven by presuppositional information encoded in determiners? An advantage of the present study over previous ones is that disambiguation was possible on the determiner itself, and not only on the noun. This makes it possible to identify immediate effects of the number information encoded in the determiner. Another advantage of the study presented here is that participants were directly addressed by the speaker. The assumption was that hearers would use all available cues given that they had to do a task for the speaker. Moreover, participants were informed that the speaker shared the same knowledge. As opposed to previous studies, participants thus had an incentive to draw the relevant inferences, and were informed of the epistemic state of hearer and speaker they needed to assume.

### Experiment

In this section, we will first provide a forecast of the experimental approach followed by a brief introduction into mouse-tracking, the method we used to answer our main questions. We will then lay out the hypotheses in more detail.

The entire experiment comprises two parts. The first part is a forced choice production task where participants are asked to produce a sentence which appropriately describes a given situation. This task has several purposes: First, we aim at showing that participants are aware of the uniqueness presupposition of the definite determiner, and therefore use it predominantly in situations where the described item is unique. Second, we aim at testing whether participants systematically use the indefinite determiner when the object they want to refer to is non-unique. Finally, the data from the production task will be used to screen participants regarding whether they have a sufficient command of the correct usage conditions of both determiners in sentence production. We will exclude participants who commit more than 20% errors, that is, who do not use determiners in the intended way or make errors with the color or object choice (these participants will be replaced with new participants).

The second part is a mouse-tracking experiment with the aim to test whether listeners can rapidly integrate potential cues about uniqueness or anti-uniqueness in a context to achieve early predictive disambiguation, even before hearing the lexically disambiguating referent noun.

#### Mouse-tracking

Mouse-tracking has become a common method in cognitive psychology, since recent studies revealed that motion trajectories can reflect underlying cognitive processes. In fact, simple hand movements offer a continuous stream of motor output and provide real-time read-outs of ongoing cognitive processes. Spivey et al. (2005) were among the first who used mouse-tracking to answer language-related questions. Participants were instructed to start a trial via moving the mouse into a start box (in the lower center of the screen) and then follow the instructions of an auditory stimulus sentence (e.g., “Click on the candle!”) while watching a picture with items in the upper left and upper right corners of the screen. In one condition, the depicted words were similar in their initial phonemes (e.g., “candle” vs. “candy”), while in the other condition they were not (e.g., “candle” vs. “summer”). The trajectories of the movements towards the correct upper corner showed an attraction of the distractor word, when both words shared the initial phonemes. Thus, during processing of the target word, competing phonological representations appear active and influence the exact way the hand moves. Similar approaches have since been applied to, for example, social cognitive questions (Freeman, Dale, & Farmer, 2011), conflict tasks (Scherbaum, Dshemuchadse, Fischer, & Goschke, 2010), the effects of irrelevant stimulus variation on action execution (Janczyk, Pfister, & Kunde, 2013), or the influence of actions consequences on action execution (Pfister, Janczyk, Wirth, Dignath, & Kunde, 2014). Furthermore, mouse-tracking has also been used in sentence verification tasks to study conversational implicatures (Sauerland, Tamura, Koizumi, & Tomlinson, 2015; Tomlinson, Bailey, & Bott, 2013), predictive disambiguation based on early intonational cues (Roettger & Stöber, 2017; Roettger & Franke, 2019), and sentence negation (Dale & Duran, 2011).

**Fig. 1:**
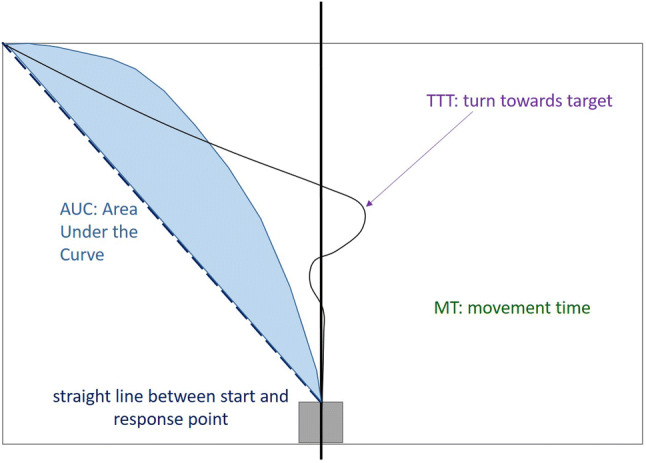
Illustration of parameters extracted from the mouse-trajectories used for further statistical analyses

Several parameters are usually extracted from the trajectories for further statistical testing. We here focus on the following parameters (see Fig. 1 for an illustration): (1) area under the curve (AUC) is the geometric area between the observed mouse-trajectory and an idealized straight line and becomes larger, the more the trajectory deviates from the straight line. According to Freeman and Ambady (2010), AUC provides a general measure of processing difficulty (Farmer, Cargill, & Spivey, 2007; Freeman, Ambady, Rule, & Johnson, 2008; Spivey, 2008). (2) Movement time (MT) is the time from stimulus onset until reaching the target box. (3) Turn towards target (TTT) is defined as the point in time when participants finally make their decision and turn towards the target without any subsequent reversals (Roettger & Franke, 2019).

In the experiment proper, participants were asked to hand a named object from a shelf to the speaker. One or two items of the named object type were present on the shelf. In addition, one or two entities of a different object type (the competitor) were present. Stimulus sentences either contained the definite or the indefinite determiner. In “early disambiguation” conditions, participants could, in principle, know which object will be referred to already upon hearing the determiner. In contrast, in “late disambiguation” conditions, this is only possible after the target object has been named. Conceivably, however, early disambiguation makes sense only if a listener knows by experience that sentences with definite and indefinite determiners are used in a felicitous way. Thus, one group of participants only encountered felicitous sentences (the reliable group), a second group was presented with infelicitous sentences as well (the unreliable group).

An advantage of using German stimuli is that we can make sure that the definite description could not be plural, as the definite determiner is marked for grammatical gender. As the plural determiner for all three genders is form-identical with the singular feminine determiner, we only used masculine and neuter nouns in our experiment, which makes the definite determiner unambiguously singular. This is not the case in English, where “the” could still be followed by plural nouns, which would make the sentence completely acceptable in non-unique scenarios. The presupposition of definite determiners is that there is a unique maximal element, which is trivially fulfilled for the extension of any plural noun (as there always must be a unique maximum) (cf. Heim, 2011). Only in combination with the singular does the definite noun phrase presuppose uniqueness of an atomic individual with the described property. It is thus possible to predict on the (singular marked) definite determiner that the reference will fail if there are only non-unique referents in German, but not in English, where plural/singular marking only becomes obvious on the noun. The general expectation is thus that participants in our experiment could use the information of number encoded in the determiner right away and make the according choices rapidly.

#### Hypotheses

The first question we ask is whether people utilize the relevant meaning components of a determiner (uniqueness and anti-uniqueness) to form expectations about the likely referent, even before this referent is lexically given (and thus disambiguated). This is supported by previous experiments showing that presupposition processing starts on the trigger itself (e.g., Tiemann et al., 2011; van Berkum et al., 1999, 2003). Thus, for the “reliable group”, we hypothesize that conditions with unequal amounts of different objects on the shelf (i.e., the early disambiguation conditions) allow faster decisions already upon encountering the determiner in comparison with those conditions with equal amounts of different objects on the shelf (i.e., the late disambiguation conditions; Hypothesis 1).

A second question is whether there are processing differences between the definite and the indefinite determiner. The three theories introduced in Sect. “Presuppositions” allow three different predictions: (1) according to the “presupposition theory”, both determiners come with their own presuppositions. In this case, the prediction would be that both the uniqueness and the anti-uniqueness presupposition are accessed equally fast and cause comparable processing difficulties. Thus, no differences are expected in this case (Hypothesis 2a). (2) According to the “anti-presupposition theory”, processing the indefinite determiner requires an initial consideration of the uniqueness presupposition of the definite determiner and its subsequent negation. This predicts more processing difficulties for the indefinite than for the definite determiner (Hypothesis 2b; see also Schneider et al., 2019). (3) Finally, the “implicature theory” assumes that the indefinite determiner activates a different type of competition, namely on the level of assertion. This would make the associated inference an implicature, which is processed more rapidly than a presupposition. Accordingly, less processing difficulties are predicted for the indefinite than for the definite determiner (Hypothesis 2c; see also Bill et al., 2018).

A final question is whether the potential early effects of information about uniqueness and anti-uniqueness (as suggested in Hypothesis 1) are affected by occasionally infelicitous uses of the determiner.

The theories spelled out above make different predictions regarding how processing of either determiner is influenced by occasional infelicitous uses. According to the anti-presupposition theory, the definite determiner comes with a lexically stored presupposition whereas the indefinite determiner’s inference is the result of pragmatic reasoning. As a consequence, the definite determiner should be less affected by infelicitous uses than the indefinite determiner given the assumption that lexical information is generally harder to overwrite. Following the presupposition theory, both determiners come with lexically encoded information regarding number. Therefore, they should be equally affected by infelicitous uses. Finally, in the case of the implicature theory, the indefinite determiner should be more affected than the definite determiner. This is because implicatures are highly context-dependent expressions, easily affected by speaker reliability (Bott & Noveck, 2004), as opposed to lexical presuppositions.^4^ These hypotheses will be referred to as Hypothesis 3.

## Method

### Participants

The intended sample size was *n* = 60. In total, data were collected from 76 people from the Tübingen (Germany) area. We excluded 14 participants that committed more than 20% errors in the production task, one participant was not a native speaker of German, and one additional participant was excluded because of technical problems during data recording (final sample: mean age = 23.6 years, 48 females, 12 males). All participants in the final sample were native speakers of German, reported normal or corrected-to-normal vision and hearing abilities, and signed written informed consent prior to data collection. Participants were randomly assigned to either the reliable group or the unreliable group of the mouse-tracking part of the experiment, with $n=30$ per group.

### Apparatus and stimuli

Stimulus presentation and response collection was controlled by a notebook connected to a TFT-screen (resolution: 1280 $\times$ 1024 px.; visible screen size: 53 $\times$ 30 cm). A standard computer mouse was used with slightly reduced cursor speed and non-linear acceleration turned off.

At the top of each screen, a shelf with ten compartments was visualized. The left- and the rightmost compartment contained either one or two objects (the same within one compartment, but different objects on the left and right side). A total of 19 different objects was used, each object in two different colors.

**Fig. 2:**
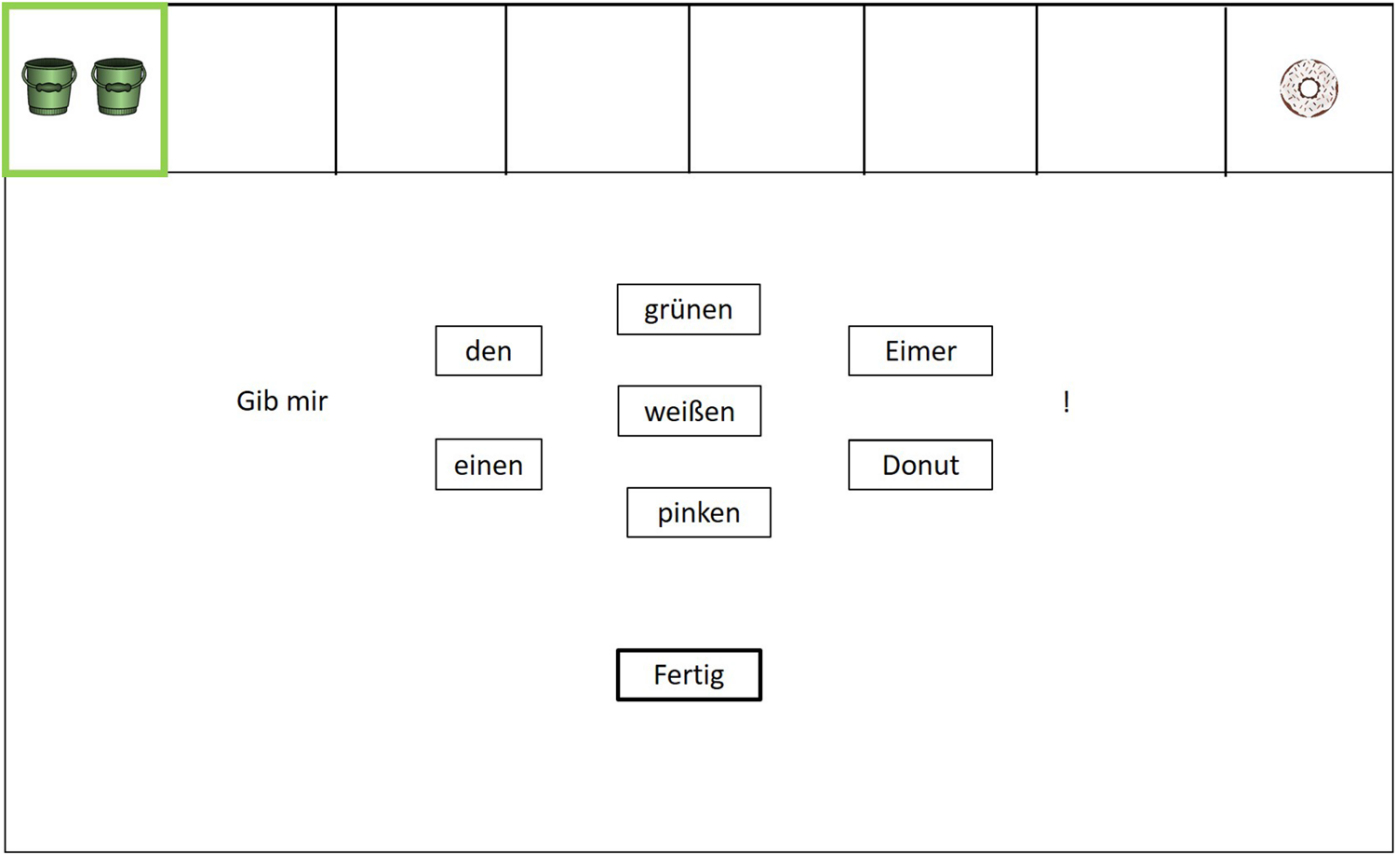
Example of the stimulus setup for a trial in the production task

During the production task, additional elements were visible on the screen on each trial (see Fig. 2). First, either the left- or the rightmost compartment was outlined in green color to indicate the relevant type of object in the current trial. Second, the German words “Gib mir” (Engl.: “Give me”) indicated the start of the to-be-completed sentence. Third, three rows of boxes contained the words the participants were to choose to complete the required sentence (left row: determiner; middle row: adjective (i.e., color of the object); right row: noun (i.e., the object)). In the production task, objects were (white or pink) donuts and (green or white) buckets. An additional box below contained the German word “Fertig” (Engl.: “Done”). Clicking on a box turned the frame bold to indicate selection.

Four different conditions of required sentences were constructed (see Table 1), resulting from combining two variables. (1) The variable *determiner* captures whether the correct determiner would be the definite or the indefinite determiner. (2) The variable *disambiguation* indicates whether an early part of the sentence (i.e., the determiner) can be used to disambiguate the sentence meaning, or only a late part (i.e., the noun). Early disambiguation is possible when different numbers of target and of the competitor are present; only a late disambiguation is possible when the same number of target and competitor is present.

**Table 1:** Summary of all four types of conditions for the production task. In the examples, we assume that the required object is located in the leftmost compartment of the shelf. (Note: determ. = determiner; disamb. = disambiguation; def. = definite; indef. = indefinite)

	determ.	disamb.	target	competitor	sentence
1	def.	early	1 bucket	2 donuts	Give me **the** green bucket
2	def.	late	1 bucket	1 donut	Give me **the** green bucket
3	indef.	early	2 buckets	1 donut	Give me **a** green bucket
4	indef.	late	2 buckets	2 donuts	Give me **a** green bucket

**Fig. 3:**
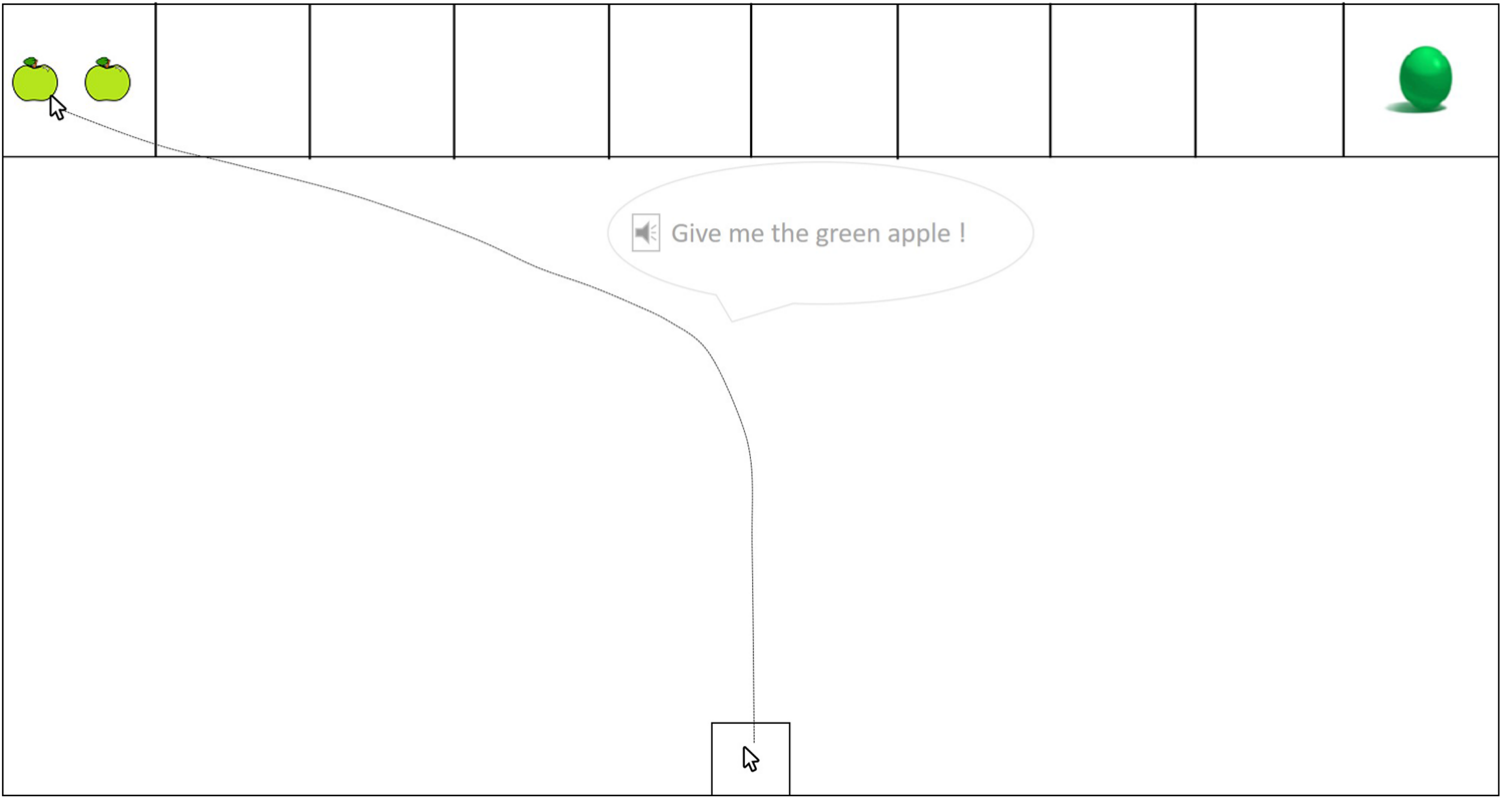
Example of the stimulus setup for a trial in the mouse-tracking task

For the mouse-tracking task, an additional box was present centrally at the bottom part of the screen as the start box (see Fig. 3). The left- and rightmost compartments of the shelf served as the response boxes. The remaining 17 objects (i.e., excluding the donut and bucket, see Table 5 in the Appendix) were used in the mouse-tracking task. In the following, we will refer to the object mentioned in the auditory stimulus sentence as the target. The other object, appearing in the opposite compartment of the shelf, will be referred to as the competitor. Some restrictions apply to the construction of the possible trials. First, the common nouns for all competitors had the same grammatical gender as those for the target, to avoid early disambiguation by this information. Second, for the same reason, targets and competitors were always presented in the same color. Third, no nouns with feminine gender were used, because the German feminine determiner “die” could also occur in combination with a plural object (e.g., “die roten Kerzen”; Engl.: “the red candles”).

Stimulus sentences as in (17) were pre-recorded and delivered via headphones. These auditory stimuli were constructed in a way to keep prosodic characteristics as constant as possible. To this end, in a first step, all sentences were recorded. Then the sentences were cut and put back together, with the same initial part (“Gib mir den/das”; Engl.: “Give me the”) for all stimuli including the definite determiner and the same initial part (“Gib mir ein/einen”; Engl.: “Give me a”) for all stimuli including the indefinite determiner. The remainder of the sentence—for example, “grünen Apfel” (Engl.: “green apple”)—was the same for definite and indefinite conditions. It was combined with each of the beginnings to keep phonological differences between sentences with definite and indefinite determiners minimal. For every second item, either the sentence with the definite determiner or the sentence with the indefinite determiner was used as the basis of cutting and pasting. An example of the setup for the mouse-tracking is given in Fig. 3.

17. Gib mir den / einen grünen Apfel!
  Give me the / a green apple!

Each relevant pairing of visual display and sentence can be classified along three variables with two levels, thus resulting in eight possible conditions (see Table 2 for a summary). (1) The variable *determiner* captures whether the sentence comprises a definite or indefinite determiner. (2) Based on the objects in the shelf, the number information carried by the determiner can either be used to disambiguate early or not; this is captured in the variable *disambiguation*: disambiguation could, in principle, happen early (on the determiner) or only late (on the noun). (3) Finally, the variable *felicity* captures whether the use of determiner and head noun was felicitous given the objects in the shelf, that is, whether the number information carried by the determiner about the head noun was actually true.

To create different instances of these relevant experimental conditions, we took the 17 objects, 5 of which were neuter and 12 were male in gender, and each was instantiated in one of two colors. We then created all possible instances of each of the eight experimental conditions by picking one object as target and another as the competitor, such that target and competitor had the same gender and the same color. Pictures instantiating the relevant experimental conditions were then created by showing one or two pictures of the target object together with one or two objects of the competitor object, depending on the requirements of the condition to be instantiated. We then created the sentence belonging to this condition, using either the definite or indefinite determiner, always including the name of the target object, of course. In this way, 138 instances of each condition were created. In the experiment, participants saw random instances of each condition, such that no participant saw the same instance of a condition twice.

**Table 2:**
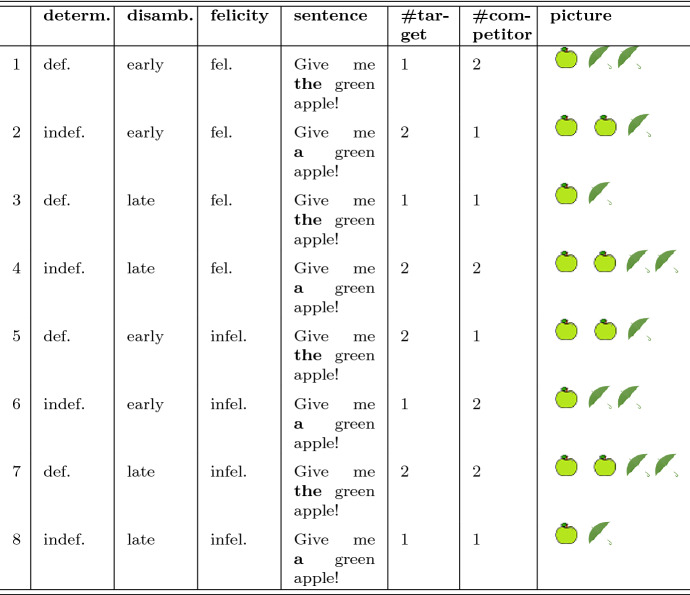
Summary of all eight possible experimental conditions for the mouse-tracking task

The actual experiment used conditions 1–6. The target in this example is always “green apple”, and “green umbrella” is the competitor. (Note: determ. = determiner; def.  = definite; indef. = indefinite; disamb. = disambiguation; fel. = felicitous; infel.  = infelicitous; # = number of)

### Task and procedure

All participants started with the production task and then performed the mouse-tracking task. For the *production task*, participants had to build a sentence to describe the item in the box, which was highlighted by a green frame. In other words, they were to provide an accurate description of the highlighted object. To this end, they were instructed to describe the picture to a second interlocutor, who views the same shelf with objects, but who is not able to see the highlighted box. In addition, participants were told that it is only possible to take one object out of the shelf.

Participants selected words with a left mouse-click within the respective boxes. When a word from all three columns was selected, a click on the “Fertig”-box initiated the next trial. Once made, a selection could not be changed. The two objects, their colors, and the different possible quantities resulted in 32 different trials, which were presented in randomized order. The position of the target (i.e., the left- or rightmost compartment of the shelf) was determined randomly for each trial with the restriction that the target appeared 16 times on each side.

The participants’ task in the mouse-tracking task was to select one of the two potential referents presented in the left- and rightmost shelf compartment according to the auditorily presented stimulus sentence. To this end, the participants were to move the mouse-cursor from the start box into the corresponding response box, that is, into the corresponding shelf compartment as soon and as fast as possible.

Each trial started with the presentation of the empty shelf and the starting box. When the mouse cursor remained within the start box for 500 ms, the target and competitor objects appeared in the shelf. From then on, participants were to start their mouse movement within 5000 ms and to finish it within 12,000 ms. Stimulus presentation was initiated when the mouse cursor was moved a minimum of 60 px. outside the start box in the upper direction, within a corridor of 60 px. horizontally centered. This was done to avoid that participants leave the start box in a diagonal direction and to ensure that they were already moving the mouse when they had to make their decision. Participants were instructed to select the correct referent as soon as possible, and to pursue a smooth movement without stops and movement direction reversals.

All participants received 48 experimental trials of each of the Conditions 1–4 (see Table 2). In these trials, the determiner was always used felicitously. Participants in the *unreliable group* received additional 48 trials of Conditions 5 and 6 (although the main analyses focus on those sentences of Conditions 1–4). Thus, the unreliable group saw more items in total than the reliable group did, and as a consequence, the experiment took slightly longer. The experimental trials were divided into four blocks of 12 trials of Conditions 1–4 (for both groups) and the additional 12 trials of Conditions 5 and 6 in the unreliable group. In sum, each block comprised 48 trials for the reliable group and 72 trials for the unreliable group, presented in random order.^5^ In half of the trials, the target appeared in the leftmost unit of the shelf; in the other half it appeared in the rightmost unit. Participants were instructed that they are interacting with another cooperative speaker who is in the same situation as they are, that is, who sees the same picture as the participants.

Prior to the experimental blocks, the experimenter demonstrated proper use of the mouse (i.e., without stopping or reversing the movement) with two trials from Conditions 1–4 each, that is, with eight trials in total. Subsequently, participants practiced the task on twelve trials. For the reliable group, these were three trials from Conditions 1–4; for the unreliable group, these were two trials from Conditions 1–6. (This procedure also ensured that participants in the unreliable group already encountered unreliable conditions.)

### Design and analyses

The independent variable of interest in the production task is the type of the determiner (definite vs. indefinite). We analyzed the percentage of infelicitous uses of the determiners, as well as erroneous choices of color and object separately. In addition, the total percentage of errors (i.e., infelicitous uses of determiners and wrong color/object choices) were analyzed. All comparisons were done with paired *t*-tests. Participants with more than 20% errors in total were replaced with new participants.

For the mouse-tracking task, erroneous trials (selection of the wrong object, initiation time too long, response too slow), trials with stops of the mouse movement (no movement within 200 ms) or backwards movements on the *y* axis (> 2 px.) were excluded from the data set first. Trajectories were then aligned to a common starting point ($x=0 ,y = 120$). Trajectories ending in the right response box were mirrored such that all trajectories ended in the left response box. Calculation of the dependent measures AUC, MT, and TTT was based on raw trajectories (Kieslich & Henninger, 2017). To remind the reader of what these measures represent: AUC is area under the curve, MT is movement time, TTT is turn towards target.^6^ To plot mean trajectories, data was time-normalized to 101 time steps. All screening and pre-processing was done by custom R-scripts and the R-package mousetrap (Kieslich & Henninger, 2017). For analyses of AUC, MT, and TTT, the data were screened for outliers, and trials were excluded if the respective value deviated more than 2.5 standard deviations from the design cell mean (calculated separately for each participant).

The main analyses focused on trials from Conditions 1–4, which were administered in both groups. Thus, mean AUC, MT, and TTT values were submitted to 2 $\times$ 2 $\times$ 2 mixed ANOVAs with determiner (definite vs. indefinite) and disambiguation (early vs. late) as repeated-measures, and group (reliable vs. unreliable) as a between-subject variable. Error percentages (based on selection of the wrong response box) were submitted to the same ANOVA to exclude trade-offs with speed-based measures (Liesefeld & Janczyk, 2019).

## Results

### Production task

Descriptively, participants used the indefinite determiner infelicitously more often, and also made more errors for color and object choice in sentences demanding the indefinite determiner (see Table 3). However, none of the differences was significant. Participants who made more than 20% errors in total were replaced with new participants. This procedure lead to exclusion of 14 participants.

**Table 3:** Percentages of infelicitous uses and errors in the production task

	Determiner		*t*-test
	Definite	Indefinite	
Determiner	4.69	5.62	*t*(59) = 0.82, *p* = 0.414, *d* = 0.11
Color	1.67	2.50	*t*(59) = 1.43, *p* = 0.159, *d* = 0.18
Object	1.46	1.67	*t*(59) = 0.44, *p* = 0.659, *d* = 0.06
Total	6.15	7.50	*t*(59) = 1.10, *p* = 0.274, *d* = 0.14

### Mouse-tracking task

**Fig. 4:**
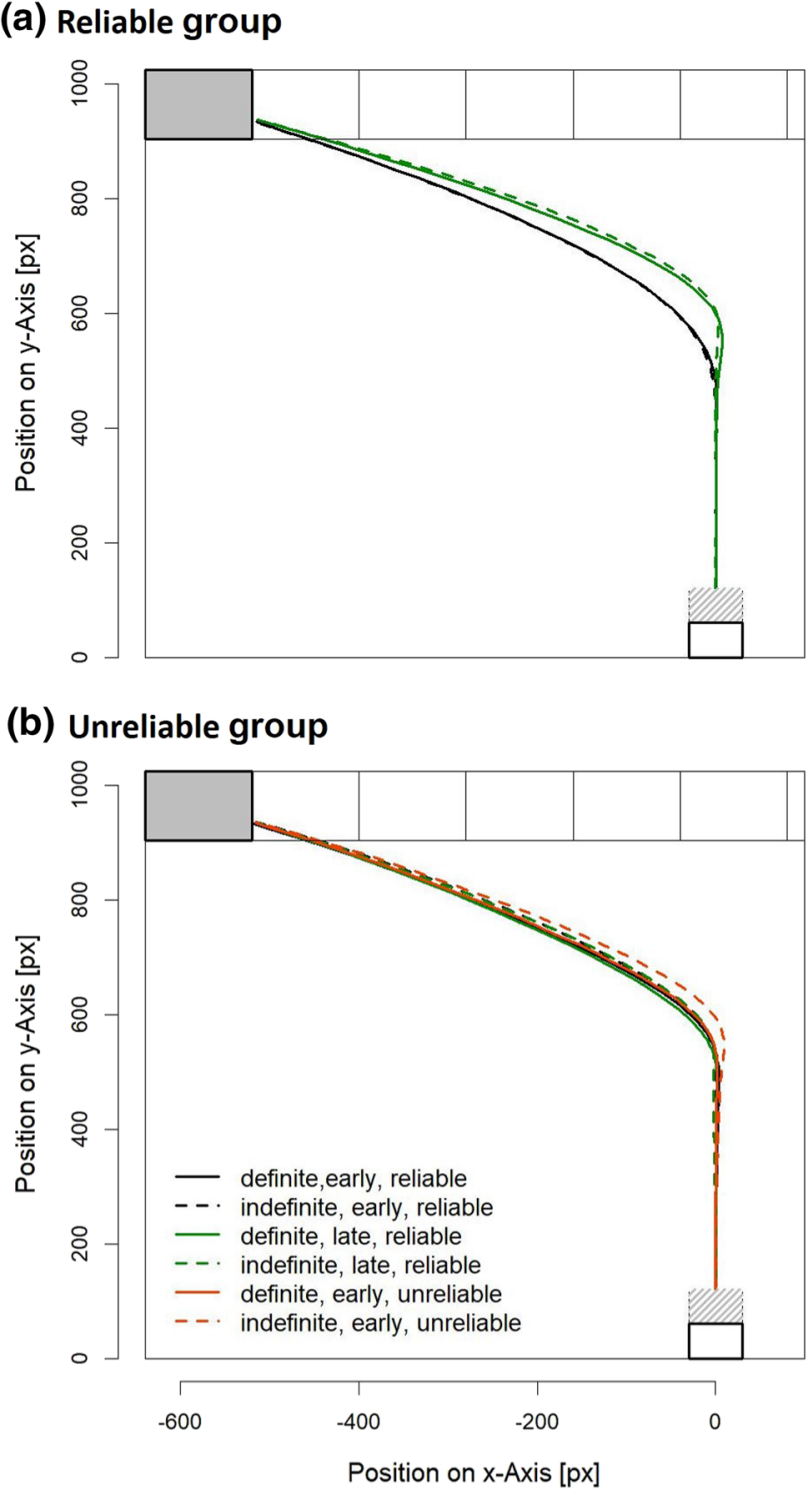
Mean trajectories of the reliable group (**a**) and the unreliable group (**b**). Note that early, unreliable conditions were only implemented in the unreliable group

**Fig. 5:**
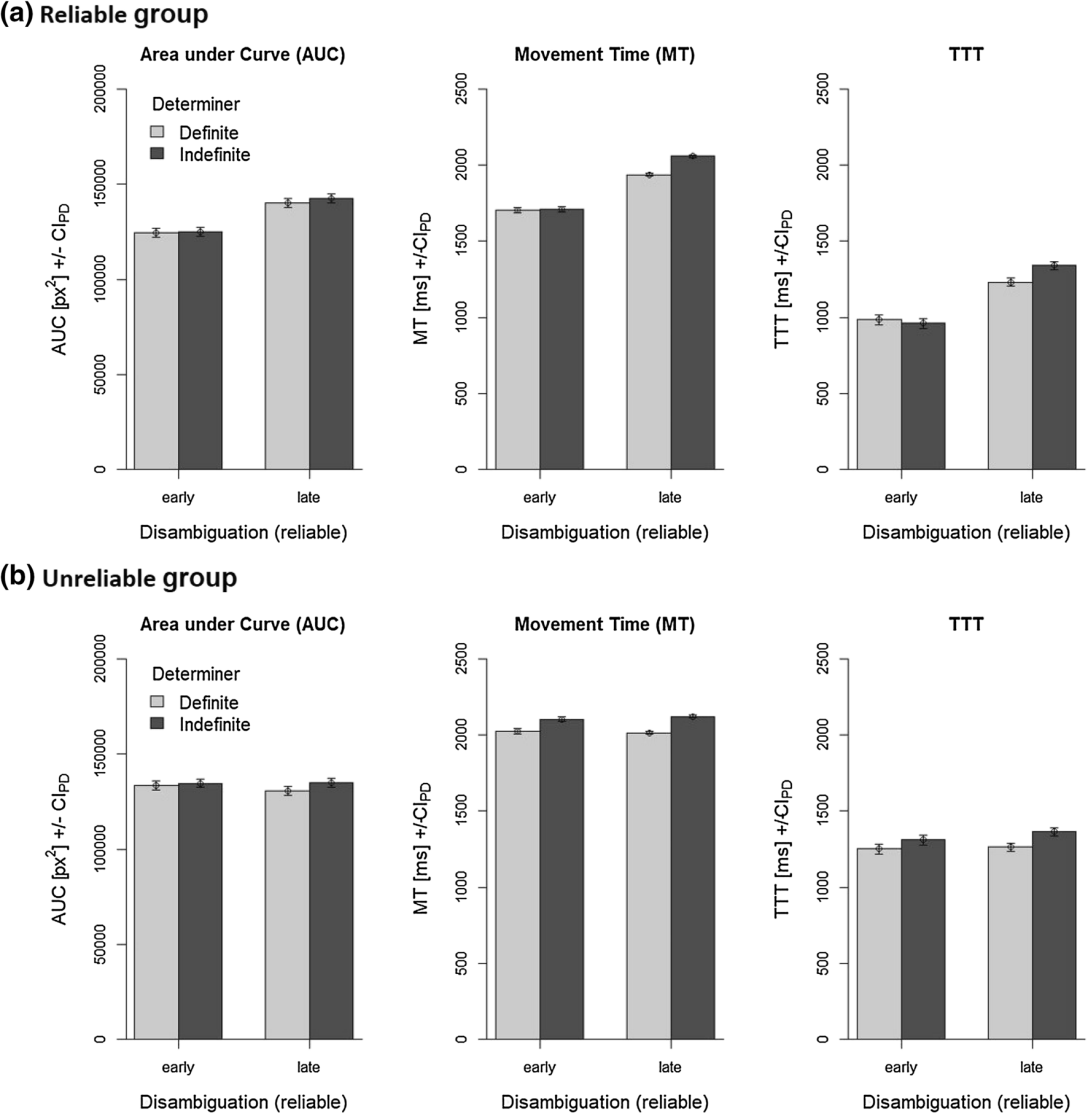
Dependent measures area under curve (AUC), movement time (MT), and turn toward target (TTT) as a function of disambiguation and determiner separately for the reliable group (**a**) and the unreliable group (**b**). Error bars are 95% confidence intervals calculated separately for each comparison of the determiners (see Pfister & Janczyk, 2013)

Mean trajectories are visualized in Fig. 4 as a function of sentence type and determiner: for the reliable group (see Panel (a)), mouse trajectories show earlier deviations into the final response locations for the early compared with the late disambiguation conditions for both types of determiners. For the unreliable group, in contrast (see Panel (b)), no such difference is readily observable. The impression thus is that participants indeed used the determiners to disambiguate sentence meaning, but only when all sentences were used felicitously, that is, in the reliable group. This impression is also reflected in AUC, MT, and TTT measures (see Fig. 5).

The ANOVA on AUC (1.57% outliers) revealed no significant main effect of group, *F*(1,58) < 0.01, *p* = .974, $\eta _p^2$ < .01. AUC was slightly larger for the indefinite determiner, *F*(1,58) = 7.21, *p* = 0.009, $\eta _p^2$ = 0.11, but determiner did not interact with either group, *F*(1,58) = 0.70, *p* = 0.406, $\eta _p^2$ = 0.01, nor disambiguation, *F*(1,58) = 1.99, *p* = 0.164, $\eta _p^2$ = 0.03. AUC was overall larger with late compared with early disambiguation, *F*(1,58) = 47.72, *p* < 0.001, $\eta _p^2$ = 0.45; however, this was mainly true for the reliable group, hence a significant interaction occurred, *F*(1,58) = 65.09, *p* < .001, $\eta _p^2$ = 0.53. The three-way interaction was not significant, *F*(1,58) = 0.07, *p* = 0.789, $\eta _p^2$ < 0.01.

Concerning MTs (1.65% outliers), all effects of the ANOVA were significant, all $F\ge$ 10.20, all *p*s $\le$ 0.002. Because this included the three-way interaction, we ran separate 2 $\times$ 2 ANOVAs for each group. For the reliable group, MTs were longer with late compared with early disambiguation, *F*(1,29) = 85.20, *p* < 0.001, $\eta _p^2$ = 0.75, and overall longer for the indefinite compared with the definite determiner, *F*(1,29) = 68.34, *p* < 0.001, $\eta _p^2$ = 0.70. This latter effect was, however, much more pronounced for late disambiguations; hence, a significant interaction, *F*(1,29) = 57.38, *p* < 0.001, $\eta _p^2$ = 0.66. For the unreliable group, MTs were comparable for both disambiguation conditions, *F*(1,29) = 0.32, *p* = 0.577, $\eta _p^2$ = 0.01, but longer for the indefinite compared with the definite determiner, *F*(1,29) = 509.02, *p* < 0.001, $\eta _p^2$ = 0.95. This effect was again (slightly) larger for late disambiguations; hence, a significant interaction, *F*(1,29) = 8.14, *p* = 0.008, $\eta _p^2$ = 0.22.

Regarding TTTs (3.01% outliers), only the interaction of group and determiner was not significant, *F*(1,58) = 3.57, *p* = 0.064, $\eta _p^2$ = 0.06. All other effects were significant, all $F\ge$ 5.01, all *p*s $\le$ 0.029. Because this included the three-way interaction, we ran separate 2 $\times$ 2 ANOVAs for each group. For the reliable group, TTTs were longer for late compared with early disambiguations, *F*(1,29) = 96.89, *p* < 0.001, $\eta _p^2$ = 0.77, and overall longer for the indefinite compared with the definite determiner, *F*(1,29) = 8.18, *p* = 0.008, $\eta _p^2$ = 0.22. This latter effect was, however, only present for late disambiguations; hence, a significant interaction, *F*(1,29) = 22.74, *p* < 0.001, $\eta _p^2$ = 0.44. For the unreliable group, TTTs were slightly longer for late compared with early disambiguations, *F*(1,29) = 6.57, *p* = 0.016, $\eta _p^2$ = 0.18, and longer for the indefinite compared with the definite determiner, *F*(1,29) = 32.73, *p* < 0.001, $\eta _p^2$ = 0.53. The interaction was not significant, *F*(1,29) = 1.77, *p* = 0.194, $\eta _p^2$ = 0.06.

Error percentages are summarized in Table 4. No effect was significant, all $F\le$ 2.56, all *p*s $\ge$ 0.115.

**Table 4:** Error percentages in the mouse-tracking task

	Group			
	Reliable		Unreliable	
	Disambiguation		Disambiguation	
Determiner	Early	Late	Early	Late
Definite	0.50	1.06	1.41	1.12
Indefinite	1.02	0.85	1.17	1.38

## Discussion

We investigated whether listeners use information about uniqueness or anti-uniqueness encoded in definite and indefinite determiners to disambiguate sentence meaning as early as possible, that is, already before the noun. Participants first performed a production task in which they chose a determiner to form a contextually adequate request for one of several visually presented objects. Subsequently, they performed a mouse-tracking task where they were to select an object according to an auditorily presented stimulus sentence.

### Summary of results

Error rates in the production task were overall low and participants generally used the determiners as expected. Thus, the results of the production task reveal that participants are aware of the uniqueness presupposition of the definite determiner, and that they associate the indefinite determiner with anti-uniqueness. As error rates were comparable for both determiners, choosing one or the other in forming contextually adequate object requests appears to be equally difficult.

For the reliable group, mouse-trajectories were affected by whether a sentence allowed for an early disambiguation on the determiner or not: AUC values were smaller and MTs and TTTs were shorter for the early compared to the late disambiguation condition. This suggests that participants use the information encoded in the determiner to disambiguate sentence meaning as soon as possible, that is, on the determiner. Further, the number information appears to be encoded in both determiners and is rapidly accessible. This result fits well with results from a study on the French language that demonstrated that gender information encoded in the determiner is also exploited immediately (Dahan et al., 2000).

However, the same was not true for the unreliable group, for which determiners were occasionally used infelicitously, and early versus late disambiguation did not affect trajectories. We conclude that exposure to infelicitous uses of determiners made participants stop using them as early cues. This suggests that deriving (anti-)uniqueness inferences must, at least in part, be a context-sensitive (pragmatic) process. In sum, the results from the mouse-tracking task yield supportive evidence for Hypotheses 1 and 3.

The results regarding Hypothesis 2 (differences between the definite and the indefinite determiner) are less clear. To start with, the data do not support Hypothesis 2c according to which the inference associated with the indefinite determiner are viewed as an implicature (Bill et al., 2018). In this case, sentences with indefinite determiners should yield less processing difficulties than sentences with definite determiners. However, no dependent variable analyzed had smaller values for the indefinite compared to the definite determiner. Considering AUC as the dependent variable, no obvious differences between the definite and the indefinite determiner are apparent, a pattern in line with Hypothesis 2a: following “the presupposition theory”, both determiners come with their own uniqueness/anti-uniqueness presupposition, and we predicted no difference in processing accordingly. Note that we also observed no significant difference in error rates between determiners in the production task.

However, not all the results are consistent with Hypothesis 2a. First, for the time-based measures MT and TTT, we observed longer times for the indefinite than for the definite determiner in the late disambiguation condition. This supports Hypothesis 2b (the anti-presupposition theory) according to which the indefinite determiner causes more processing difficulties than the definite determiner due to the additional reasoning processes involved in deriving its inference. It is noteworthy that, qualitatively, we obtained the same pattern for the early disambiguation condition within the unreliable group. Thus, when the information encoded in the determiner is no longer used for immediate disambiguation, the indefinite determiner induces more processing difficulties than the definite determiner. Second, that the determiners both encode presuppositions is unlikely given the results regarding Hypothesis 3. Presuppositions should be harder to override given that they are assumed to be lexical information, whereas implicatures are (to a certain degree) optional inferences which participants can be trained to ignore (Bott & Noveck, 2004). In sum, it appears that the results are partly in line with Hypothesis 2b and thus support the anti-presupposition theory (see also Schneider et al., 2019), although we contend that some aspects of the data are in line with Hypothesis 2a as well.

### Theoretical implications

According to the anti-presupposition theory, processing indefinite determiners involves an initial consideration of the uniqueness presupposition of the definite determiner (its alternative) and its subsequent negation. This should then result in more processing difficulties for the indefinite determiner compared to the definite determiner (see also Schneider et al., 2019). Why, then, was this pattern much more pronounced for the late compared to the early disambiguation condition, especially in the reliable group? One possible explanation is that in the late disambiguation condition of the reliable group, the indefinite determiner does not reduce uncertainty about which of the two non-unique objects the speaker refers to. This problem does not occur for the definite determiner because the two different objects on the shelf are unique, respectively. In the early disambiguation condition, this consideration may have played less of a role given that decisions were made already before the information from the subsequent disambiguating noun was processed. A further alternative explanation will be discussed in Sect. “Limitations and future work”.

Moreover, it is in line with Hypothesis 2b that early cues are not used anymore for the indefinite determiner in the unreliable group. Given that the inference is a result of pragmatic reasoning over alternatives, it should be affected by reliability of the speaker (Rouillard & Schwarz, 2017). It remains surprising and unexplained, however, why this is not different for the definite determiner, which should be stronger in meaning according to Hypothesis 2b.

The observed pattern of performance with both definite and indefinite determiners may also partially result from particular properties of the experimental setup. From our experimental setup, it was likely clear that (1) definite and indefinite determiners are in competition, (2) the information given by the speaker was crucial to fulfill the task, and (3) the number of objects played a role. These factors combined may have led to a more parallel treatment of the two determiners than predicted by the anti-presupposition theory. In particular, the experimental setup differs from previous work in that the speaker directly addressed the participants with an order (“Gib mir...!”, Engl.: “Give me ...!”). Previous experiments either asked people to evaluate statements with definite and indefinite determiners by choosing a picture fitting their interpretation (Bade & Schwarz, 2019a) or by judging sentences as true or false (Schneider et al., 2019). It was unclear in these earlier studies who the speaker was and what his/her knowledge state was. Furthermore, it was underspecified what consequences, especially for the speaker, the participants' decision had in these experiments. These factors may have contributed to the lower percentage (around 30%) to which participants actually drew the inference associated with the indefinite determiner compared to the definite determiner (92%) in Bade and Schwarz (2019a). In other words, participants did not rely on the information encoded in the indefinite determiner for their choices. The reason for this may be uncertainty about which interpretation of the indefinite determiner was intended in the experiment, given that it is ambiguous between referential and quantificational uses. This consideration may have given rise to the clear differences between the two determiners observed with eye-tracking by Bade and Schwarz (2019a). In contrast, participants in the present experiment had a clearer idea of the goals and knowledge of the speaker, and that may have driven them to use the inferences of both determiners as early as possible to predict eventual sentence meaning. Especially in the reliable group, the use of the indefinite determiner as referring to non-unique objects was very clear and unambiguous. As a result, the anti-uniqueness inference may have become more salient and thus less easily distinguishable from the presupposition of the definite determiner in processing. Further support for this view comes from a follow-up experiment of Bade and Schwarz (2019a) reported in Bade and Schwarz (2019b) which suggests that (1) target choices for the indefinite determiner are boosted by exposure to the alternative (the definite determiner) and (2) if the anti-uniqueness inferences can reliably be drawn, the processing patterns of definite and indefinite determiners do not differ.

A further factor that may have helped in stabilizing the anti-uniqueness inference associated with the indefinite determiner is the production task that preceded the mouse-tracking task. With the production task, we evaluated whether participants are aware of the (anti-)uniqueness of determiners. Participants that did not reliably use the number information were replaced. Conceivably, the production task has already shifted participants’ attention to the inferences associated with both determiners and presented them as alternatives. In line with this, Bade and Schwarz (2019b) demonstrated that this affects the percentage of anti-uniqueness inferences. The authors varied the order of comprehension and production blocks between two groups. The choice of target pictures (with non-unique objects) for the indefinite determiner was much higher when the production block preceded the comprehension block than when it only followed the comprehension block. Bade and Schwarz thus hypothesized that making the alternatives explicit (with the production task) makes the anti-uniqueness inference more salient. Similar sensitivity to salient alternatives have been observed for scalar implicatures (e.g., Franke, 2014; Degen & Tanenhaus, 2015).

In sum, both (1) having very clear unambiguous uses of the indefinite determiner in the comprehension task and (2) presenting the definite determiner as an alternative in the preceding production task may have stabilized the anti-uniqueness inference and thus blurred the distinction between definite and indefinite determiners in the current experiment.

### Limitations and future work

One clear limitation of the present study is that it focuses only on determiners in only one language (German). Given that determiner systems show high variability cross-linguistically, it would be unwarranted to draw very strong conclusions from our findings for the processing of (indefinite) determiners in general. This is especially the case given that the indefinite determiner in German, as in many other languages, can fulfill more than one function, for example, can refer to specific objects, can be an existential quantifier, or can be a numeral. The interpretation of the indefinite determiner cross-linguistically may also depend on what kind of indefinite competitors the language offers. German, for example, has the free choice item “irgendein” (Engl.: “anyone”) as an alternative, English has “some” and the numeral “one”. Given the complexity of the empirical situation, it is unclear whether all uses of the indefinite determiner (and all indefinites) are associated with the inference of interest here, and what additional factors may play a role. To test the predictions of the anti-presupposition theory, it may thus be worthwhile to test more phenomena it has been applied to (e.g., mood, tense, number, competition between “all” and “both”) to avoid this additional complexity.

A potential problem for interpreting differences between determiners results from the fact that the sequence of the indefinite determiner and the following adjective is longer than the sequence of the definite determiner and the following adjective (693 ms vs. 575 ms on average). This is because the two determiners in the German accusative differ in number of letters and syllables when the noun is masculine. Additionally, the corresponding adjective differs due to declension. This may also explain differences between both determiners for the time-based measures MT and TTT in the late disambiguation conditions, where participants have to wait until the onset of the noun to make their decision. However, if this was the only explanation, one would expect this difference to be present in all conditions, including the early disambiguation condition within the reliable group: conceivably, participants need to process the whole determiner for its identification, and this should also differ in duration. Consequently, the same results would be expected as well. Admittedly, we cannot exclude that the mere onset of the determiner sufficed to distinguish both determiners in this particular condition. In this case, results should indeed be similar for both determiners. Accordingly, we should also qualify the conclusions made above and we concur that this would favor Hypothesis 2a more than Hypothesis 2b. On the basis of our data, we cannot clearly decide on this issue. One way to address this issue is to design a similar experiment in the English language, where the definite determiner “the” is not shorter compared to the indefinite determiner “a” and the adjective is morphologically equally complex. Finally, however, it should be noted that the important differences between the early and late disambiguation condition are not affected by this potential problem.

An interesting question which the present data cannot answer is whether participants learn to use cues associated with determiners during the experiment when they are exposed to reliable uses or whether participants use such cues from the very beginning, but “unlearn” to use them when confronted with infelicitous uses (i.e., in the unreliable group). It is unclear from the current experiment what role, if any, the production task played in this (un)learning. Clearly, participants’ attention was already drawn to the fact that number information encoded in the determiners, as well as competition between them, may play a role. To tackle this issue, future research should consider to either not include a production task, or apply this task before or after the main experiment in two separate groups. If exposure to alternatives does play a role, this should affect the processing of determiners. Moreover, to address the issue of (un)learning, the number and order of infelicitous/felicitous uses of determiners could be manipulated in follow-up studies. If number information is encoded in the lexicon for both determiners, then a gradient unlearning effect should be observed for both of them.

## Conclusion

In sum, it appears that the uniqueness and anti-uniqueness inferences associated with definite and indefinite determiners, respectively, are used rapidly to disambiguate sentence meaning and to make contingent decisions. The robustness of this is, however, affected by occasional infelicitous uses of determiners. We find that it is thus important to consider the reliability of the speaker when investigating these inferences. Regarding differences between determiners, our results are less conclusive and we refrain from drawing strong conclusions. However, the conclusion that participants used the information of the determiners to disambiguate sentence meaning as soon as possible is not undermined by this.

## Supplementary material

A preregistration report including the hypotheses, the planned design of the experiment, the stimulus material, and the planned analyses was uploaded OSF prior to data collection (2018-08-03 01:53 PM; https://osf.io/aym9p/).

### Electronic supplementary material

Below is the link to the electronic supplementary material.

Supplementary material 1 (pdf 161 KB)

## Figures and Tables

**Table 5:**
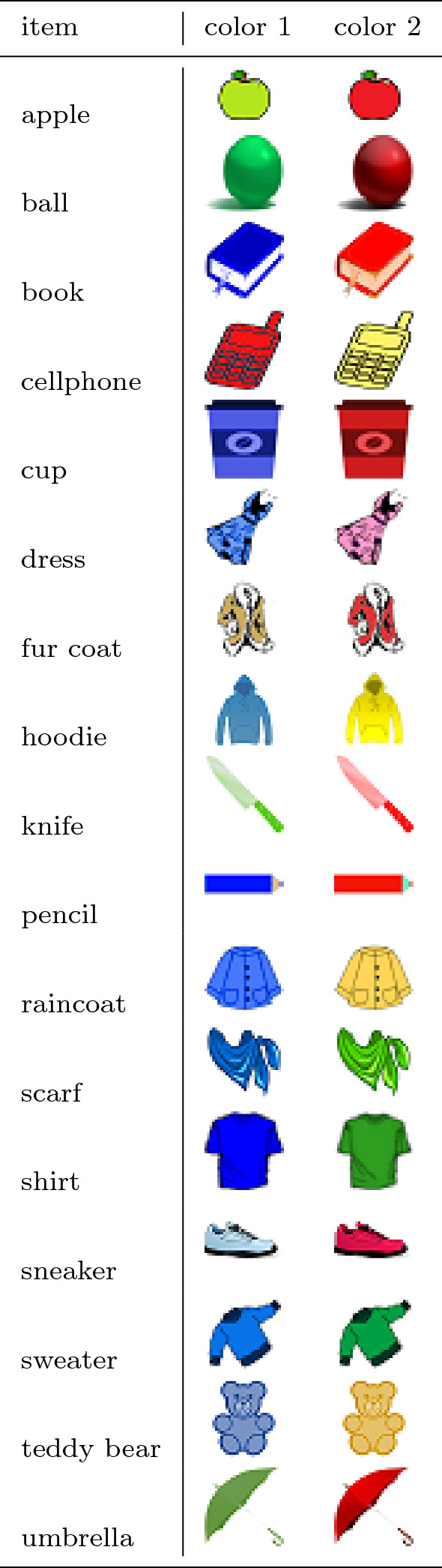
Overview of the items employed in the mouse-tracking task

Each item is depicted in its two colors

## Footnotes

1 Some authors use the term “non-uniqueness” instead of “anti-uniqueness” to refer to this effect of the indefinite determiner (Heim 1991; Sauerland, 2008; but see, e.g., Alonso-Ovalle, Menndez-Benito, & Schwarz, 2011, using the term “anti-uniqueness”). We use the term “anti-uniqueness” to describe the same phenomenon as discussed by these authors, and do not mean to imply any theoretical distinction. Importantly, we do not assume Barker’s more specialized definition of “anti-uniqueness” as definite determiners being blocked in partitive constructions (e.g., one of John’s friends; Barker, 1998).

2 We are aware that the example is outdated, as nowadays gay couples frequently adopt children, which seems to lead to language change, especially in lexical fields dealing with family concepts. We think that the example still illustrates the relevant point well though, if we imagine the noun phrase to refer to the biological father, not the person(s) fulfilling the role of a father.

3 The authors describe the N400 effect as a detection of a semantic mismatch (see, e.g., Kutas, Petten, & Kluender, 2006) and the P600 as an index for a subsequent reanalysis process (see, e.g., Kuperberg, 2007).

4 A preregistration report including the hypotheses, the planned design of the experiment, the stimulus material, and the planned analyses is available at OSF: https://osf.io/aym9p/. Parts of the preregistration were written in past tense, as if the text were a method section proper. We regret that this is somewhat unfortunate but confirm that data were collected only after posting the preregistration.

5 This is a slight deviation from the pre-registered procedure, because we decided to use unreliable items of Condition 5 and 6 equally often as the reliable items in Condition 1 and 2, and, therefore, increased the number of items in Conditions 5 and 6.

6 In the preregistration report, we also included an analysis of an additional trajectory measure—X_neg_—to compare reliable versus unreliable conditions. We did not include this analysis in the main text, because the observed data pattern does not match with the originally formulated hypothesis. The hypothesis and results are reported in an Online Supplement.
