# Supplementary material for: Presuppositions of determiners are immediately used to disambiguate utterance meaning: A mouse-tracking study on the German language
Source: Psychol Res. 2020 Apr 4;85(3):1348–66. doi: 10.1007/s00426-020-01302-7 (PMC8049927; doi:10.1007/s00426-020-01302-7)
Supplement: Supplementary file 1 — Supplementary material 1 (pdf 161 KB) [file 426_2020_1302_MOESM1_ESM.pdf]

**Noname manuscript No.**
(will be inserted by the editor)

# Online Supplement.

## Presuppositions of determiners are immediately used to disambiguate utterance meaning

**Cosima Schneider, Nadine Bade, Michael Franke, & Markus Janczyk**

Received: date / Accepted: date

## 1 Analysis of $X_{neg}$ values

In the preregistration report we originally formulated hypotheses also about $X_{neg}$ values. Because the empirical results do not fit the predicted patterns, we did not include the respective analysis in the main manuscript. For completeness, we provide the analysis in this online supplement.

The ANOVA on $X_{neg}$ (3.61% outlier) revealed no significant main effect of disambiguation, $F(1{,}58) = 0.31$, $p = .579$, $\eta_p^2 = .01$, nor did we observe a main effect of determiner $F(1{,}58) = 3.64$, $p = .061$, $\eta_p^2 = .06$. The interaction of disambiguation and determiner reached significance $F(1{,}58) = 6.56$, $p = .013$, $\eta_p^2 = .10$.

C. Schneider
University of Tübingen Schleichstrasse 4, 72076 Tübingen
Tel.: +49 (0)7071 - 29 78359
E-mail: cosima.schneider@uni-tuebingen.de

N. Bade
University of Tübingen Nauklerstrasse 35, 72074 Tübingen
Tel.: +49 7071 29 77434
E-mail: nadine.bade@uni-tuebingen.de

M. Franke
Osnabrück University Wachsbleiche 27, 49090 Osnabrück
Tel.: +49 541 969-3370
Fax: +49 541 969-3374
E-mail: michael.franke@uni-osnabrueck.de

M. Janczyk
University of Bremen Hochschulring 18 (Cognium), 28359 Bremen
Tel.: +49 (0)421 - 218-68720
E-mail: janczyk@uni-bremen.de

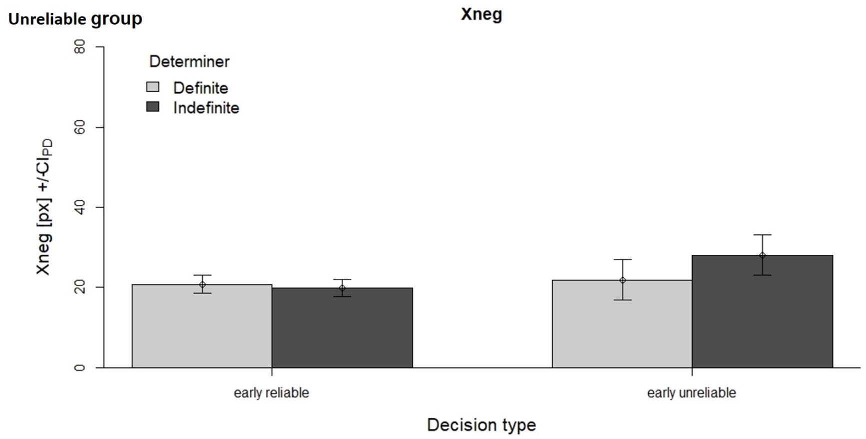


Fig. 1: Dependent measure $X_{neg}$ as a function of disambiguation (early reliable vs. early unreliable) and determiner (definite vs. indefinite) for the unreliable group. Error bars are 95%-CIs calculated separately for each comparison of the determiners (see Pfister & Janczyk, 2013).
